# IscR of *Rhodobacter sphaeroides* functions as repressor of genes for iron-sulfur metabolism and represents a new type of iron-sulfur-binding protein

**DOI:** 10.1002/mbo3.279

**Published:** 2015-08-01

**Authors:** Bernhard Remes, Benjamin D Eisenhardt, Vasundara Srinivasan, Gabriele Klug

**Affiliations:** 1 Institut für Mikrobiologie und Molekularbiologie, IFZ, Justus-Liebig-Universität35392, Giessen, Germany; 2 LOEWE-Zentrum für Synthetische Mikrobiologie, Philipps Universität Marburg35043, Marburg, Germany

**Keywords:** Fe–S proteins, iron, Iron-Rhodo-box, iron-sulfur cluster, IscR, *Rhodobacter sphaeroides*

## Abstract

IscR proteins are known as transcriptional regulators for Fe–S biogenesis. In the facultatively phototrophic bacterium, *Rhodobacter sphaeroides* IscR is the product of the first gene in the *isc*-*suf* operon. A major role of IscR in *R. sphaeroides* iron-dependent regulation was suggested in a bioinformatic study (Rodionov et al., *PLoS Comput Biol* 2:e163, 2006), which predicted a binding site in the upstream regions of several iron uptake genes, named Iron-Rhodo-box. Most known IscR proteins have Fe–S clusters featuring (Cys)_3_(His)_1_ ligation. However, IscR proteins from *Rhodobacteraceae* harbor only a single-Cys residue and it was considered unlikely that they can ligate an Fe–S cluster. In this study, the role of *R. sphaeroides* IscR as transcriptional regulator and sensor of the Fe–S cluster status of the cell was analyzed. A mutant lacking IscR is more impaired in growth under iron limitation than the wild-type and exhibits significantly increased ROS levels in iron-replete and iron-deplete conditions. Expression studies reveal that *R. sphaeroides* IscR in its cluster-bound form functions as transcriptional repressor of genes involved in iron metabolism by direct binding to the promoter region of genes preceded by the motif. A total of 110 genes are directly or indirectly affected by IscR. Furthermore, IscR possesses a unique Fe–S cluster ligation scheme with only a single cysteine involved.

## Introduction

Iron-sulfur (Fe–S) clusters are ensembles of iron and sulfide centers. The most abundant types of Fe–S clusters found in nature are the rhombic [2Fe–2S] and the cubane [4Fe–4S] clusters which contain either ferrous (Fe^2+^) or ferric (Fe^3+^) iron and sulfide (S^2−^) (Drennan and Peters 2003; Johnson et al. 2005). These clusters are typically attached to their protein partners, called Fe–S proteins, via their iron atoms, which are preferentially bound to the sulfur atoms in the cysteine residues of the peptide backbone. However, an interesting feature of Fe–S clusters is the broad diversity of ligands that the iron atoms can ligate. There are several examples of nitrogen coordination, provided by histidine or arginine residues (Peters et al. 1998; Nicolet et al. 1999; Berkovitch et al. 2004), and oxygen coordination, from aspartate, glutamine or tyrosine (Calzolai et al. 1995; Dobritzsch et al. 2001). Furthermore, there are examples of coordination by exogenous ligands, such as water, enzyme substrates or cofactors (such as *S*-adenosylmethionine) (Flint and Allen 1996; Cheek and Broderick 2001; Berkovitch et al. 2004; Fontecave 2006).

Although Fe–S clusters are easily assembled in vitro with inorganic iron and sulfur sources in a reductive environment (Malkin and Rabinowitz 1966), the in vivo situation is more complex and requires Fe–S biogenesis systems. Three different types of bacterial Fe–S biogenesis systems, Isc (iron-sulfur cluster), Suf (sulfur formation) and Nif (nitrogen fixation) have been extensively characterized (Fontecave 2006; Py and Barras 2010). Such Fe–S assembly systems are essential for virtually all living organisms and are important for the activity of many enzymes involved in diverse cellular processes, including respiration, DNA synthesis and repair, gene regulation, RNA modification, and nitrogen and carbon metabolism (Py and Barras 2010). Thanks to their chemical versatility, Fe–S clusters can act as catalysts or redox sensors and are known or predicted to be used by a large number of proteins (e.g. over 150 in *Escherichia coli*; Py and Barras 2010). However, the increase in oxygen after the emergence of oxygenic photosynthesis created a threat to Fe–S proteins and, consequently, to the organisms relying on them (Imlay 2006). In particular, reactive oxygen species (ROS) cause destabilization of Fe–S cluster, leading to release of Fe^2+^ ions that in turn fuel ROS production by Fenton chemistry. Furthermore, iron switches from the soluble (0.1 mol/L at pH 7.0) ferrous state to the extremely insoluble (10^−18^ mol/L at pH 7.0) ferric form (Andrews et al. 2003). So, in oxic conditions, iron is both poorly available and potentially toxic. Therefore, bacteria have evolved mechanisms to maintain a precise intracellular iron concentration, including iron storage proteins and a general ferric iron buffering system (such as Fe–S cluster). Several regulators of iron metabolism have been extensively investigated in bacteria. In *E. coli* and in many other bacteria the Fur protein is a major regulator for iron-dependent gene expression (reviewed in e.g. Hantke 2001). Iron regulation in *α*-proteobacteria mainly occurs by regulators different from this type of Fur protein, namely by Irr, Fur/Mur, RirA, or IscR (Johnston et al. 2007; Peuser et al. 2012). In *Rhodobacter capsulatus*, the LysR-type transcriptional regulator HbrL is a crucial regulator for the control and coupling of heme synthesis with iron homeostasis (Zappa and Bauer 2013). While no RirA homolog is found in *Rhodobacteraceae*, previous data revealed that in *Rhodobacter sphaeroides*, neither Fur/Mur nor Irr appears as a master regulator of iron homeostasis (Peuser et al. 2011, 2012). Whether putative HbrL homologs play an important role in *R. sphaeroides* remains to be elucidated.

In Rodionov et al. (2006) identified a highly conserved 19-bp palindromic signal, which was termed Iron-Rhodo-box. This motif occurs in upstream regions of most iron uptake and storage genes in all 12 at that time available genome sequences of the *Rhodobacteraceae* group. The Iron-Rhodo-box motif has a significant similarity to the RirA-box motif in the Rhizobiales. However, the absence of a RirA homolog suggests that another transcription factor mediates the global control of iron transport genes. Rodionov et al. (2006) therefore hypothesized a potential major role of IscR in *R. sphaeroides* iron-dependent gene regulation. IscR belongs to the Rrf2 superfamily of transcriptional regulators and contains a helix-turn-helix DNA-binding domain. The Rrf2 family members are not well characterized in *α*-proteobacteria, but the presence of conserved Cys residues in several of them suggests that a subset of these proteins may ligate Fe–S clusters. In many organisms, IscR acts as both, a sensor of cellular Fe–S level and as a global transcriptional regulator for Fe–S biogenesis (Giel et al. 2006; Lee et al. 2008; Giel et al. 2013). Genome-wide transcript profiling showed that IscR regulates expression of at least 40 genes in *E. coli*, among which are the *isc* operon encoding IscR itself and the Fe–S cluster biogenesis genes (*suf* genes) (Schwartz et al. 2001; Giel et al. 2006). Other regulated genes encode both, Fe–S proteins as well as non-Fe–S proteins, suggesting an important role of IscR as global regulator (Giel et al. 2006; Loiseau et al. 2007; Angelini et al. 2008; Wu and Outten 2009). Furthermore, IscR acts as a sensor of the cellular demands for Fe–S cluster biogenesis (Fleischhacker et al. 2012). Most Fe–S proteins ligate Fe–S clusters via four Cys, while IscR proteins typically have Fe–S clusters featuring (Cys)_3_(His)_1_ ligation (Fleischhacker et al. 2012). In *E. coli*, IscR exists in two major forms: apo-IscR lacks the Fe–S cluster while the holo-protein is ligated to the Fe–S cluster. Apo-IscR signals that the cell is in need of Fe–S clusters and derepresses transcription of the *isc* operon. On the other hand, holo-IscR signals that other Fe–S proteins are momentarily oversaturated and blocks the transcription of the *isc* operon (Yeo et al. 2006). Since IscR only contains three Cys residues, IscR exhibits a decreased affinity for Fe–S cluster and is therefore only able to bind the clusters when other proteins do not require them (Schwartz et al. 2001). According to this, upon oxidative stress conditions, the Fe–S of IscR is likely one of the first clusters destroyed. Recent studies have shown that in *E. coli* all three conserved Cys residues are essential for the formation of the holo-protein (Yeo et al. 2006; Nesbit et al. 2009). Interestingly, the IscR proteins from *Rhodobacteraceae* harbor only a single-Cys residue. This difference in the primary structure of *iscR* raised the possibility that IscR proteins in *Rhodobacteraceae* cannot ligate an Fe–S cluster.

*Rhodobacter sphaeroides* is a facultative phototrophic bacterium that forms photosynthetic complexes at low oxygen tension or anoxic conditions. A global transcriptome analysis in the background of a ∆*iscR* strain revealed that IscR functions as transcriptional repressor of genes preceded by a specific DNA-binding motif (Iron-Rhodo-box). Furthermore, we confirmed that despite the marked differences in sequence IscR from *R. sphaeroides* coordinates an iron sulfur center and provide first hints to amino acids involved in this ligation.

## Materials and Methods

All strains, plasmids, and oligonucleotides used in this study are listed in Tables S1–S3 of the supplementary data.

### Bacterial strains and growth conditions

*Escherichia coli* strains were grown in Luria–Bertani medium at 37°C with shaking (180 rpm) or on solid growth medium, which contained 1.6% (w/v) agar. *Rhodobacter sphaeroides* strains were cultivated at 32°C in 50-mL Erlenmeyer flasks containing 40 mL malate minimal medium (Remes et al. 2014) with continuous shaking at 140 rpm, resulting in a constant dissolved oxygen concentration of ∼25–30 *μ*mol/L during the exponential phase. Conditions of iron limitation were achieved as described previously (Peuser et al. 2011; Remes et al. 2014). When required, antibiotics were added to liquid or solid growth media at the following concentrations: spectinomycin (10 *μ*g mL^−1^); kanamycin (25 *μ*g mL^−1^); tetracycline (2 *μ*g mL^−1^) (for *R. sphaeroides*); kanamycin (25 *μ*g mL^−1^); and tetracycline (20 *μ*g mL^−1^) (for *E. coli*).

### Construction of a *R. sphaeroides iscR* deletion mutant

*Rhodobacter sphaeroides* strain 2.4.1Δ*iscR* was generated by transferring the suicide plasmid pPHU2.4.1Δ*iscR*:Sp into *R. sphaeroides* 2.4.1, and screening for insertion of the spectinomycin resistance cassette into the chromosome by homologous recombination. Briefly, parts of the *iscR* gene (RSP_0443) of *R. sphaeroides*, together with upstream and downstream sequences, were amplified by polymerase chain reaction (PCR) using oligonucleotides 0443_upA/0443_upB and 0443_downA/0443_downB. The amplified PCR fragments were cloned into the EcoRI-BamHI and BamHI-HindIII sites of the suicide plasmid pPHU281 (Hubner et al. 1991), generating the plasmid pPHU2.4.1Δ*iscR*. A 2.0 kb BamHI fragment containing the spectinomycin cassette from pHP45Ω (Prentki et al. 1991) was inserted into the BamHI site of pPHU2.4.1Δ*iscR* to generate pPHU2.4.1Δ*iscR*::Sp. This plasmid was transferred into *E. coli* strain S17-1 and diparentally conjugated into *R. sphaeroides* 2.4.1 wild-type strain. Conjugants were selected on malate minimal salt agar plates containing spectinomycin. By insertion of the spectinomycin cassette, 287 bp of the 468 bp *R. sphaeroides iscR* gene were deleted.

### Complementation of the *R. sphaeroides* deletion mutant 2.4.1∆*iscR*

For complementation of the *iscR* deletion mutant of *R. sphaeroides* a 639 bp PCR fragment containing the entire *iscR* gene along with 104 bp of the upstream region and 74 bp of the downstream region was amplified by using the oligonucleotides IscR_complA and IscR_complB (Table S3). Following digestion with BamHI and XbaI, the fragment was cloned into the corresponding sites of pBBR1-MCS-2, resulting in plasmid pBBR*iscR*. To complement the *iscR* deletion in the wild-type strain 2.4.1, the plasmid pBBR*iscR* was transferred into *E. coli* S17-1 and conjugated into the 2.4.1∆*iscR* strain by biparental conjugation.

### Cloning and expression of recombinant IscR protein from *R. sphaeroides*

Oligonucleotides IscR-His_fwd and IscR-His_rev (Table S3) were used for amplifying the coding region of *iscR*. The purified 497-bp PCR product was digested with BamHI and HindIII and ligated into the pQE30 cloning vector (Qiagen, Hilden, Germany) to yield plasmid pQE30::*iscR*. This plasmid was transformed into *E. coli* M15 cells and overexpressed as described earlier (Rische and Klug 2012). Aliquots of the fractions were separated by 10% sodiumdodecyl sulfate polyacrylamide gel electrophoresis (SDS-PAGE). After visualization with silver staining, the protein was determined as pure, since there was only one major band visible.

For site-directed mutagenesis of residues in IscR the plasmid pQE30::*iscR* was used as PCR template. Mutations were inserted with the following primers by inverse PCR: IscR_H93A_A, IscR_H93A_B, IscR_H121A_A, IscR_H121A_B, IscR_H127A_A, IscR_H127A_B, IscR_C142A_A, IscR_C142A_B, IscR_P143A_A, and IscR_P143A _B (Table S3). IscR_H93A carries a mutation at position 277–278 (CA to GC), IscR_H121A carries a mutation at position 361–362 (CA to GC), IscR_H127A carries a mutation at position 389–390 (CA to GC), IscR_C142A carries a mutation at position 424–425 (TG to GC), and IscR_P143A carries a mutation at position 427 (C to G). The mutated clones were selected and confirmed for mutations by nucleotide sequencing. After transformation with *E. coli* S17-1, the resulting plasmids were transferred to *R. sphaeroides* by diparental conjugation, yielding the corresponding reporter strains (Table S1).

### Reconstitution of iron-sulfur clusters in IscR

The chemical reconstitution of IscR was performed after Ni-NTA purification with a 100-*μ*mol/L protein solution under strictly anaerobic conditions in a Coy anaerobic chamber. Reconstitution was performed as described elsewhere (Fluhe et al. 2012). Incubation overnight in ammonium iron citrate and lithium sulfide resulted in a dark brown solution.

### Construction of a *cfp* reporter system

For construction of the fluorescence based in vivo reporter system we used a vector system designed for fluorescence based in vivo localization studies. Therefore we originally inserted the multiple cloning site (MCS) of pET28(a) (Novagen, Darmstadt, Germany), by use of the restriction sites XbaI and XhoI, into the broad host range vector pBBR1MCS2 (Kovach et al. 1995) using the same restriction sites. Thereby a ribosomal-binding site (RBS), His-tag and thrombin cleavage site were transferred from pET28(a) to pBBR1MCS2 and the derived vectors were renamed to pBE (B = pBBR1MCS2, E = pET28). A regulative DNA element for strong constitutive expression (RSP_4352 upstream sequence, see Mank et al. 2012) was inserted with XbaI, 5′to the RBS (pBE4352). A first *eCFP* DNA fragment without stop codon was inserted with the help of NdeI and EcoRI restriction sites (pBE4352::eCFP), a second *eCFP* DNA fragment with stop codon was inserted by EcoRI and HindIII 3′to the RBS (pBE4352::eCFP:eCFP). The exchange of one of the present *eCFP* fragments with a sequence of interest, the exchange of the constitutive promoter by a promoter of interest, or a constitutive over expression of N- or C-terminally tagged eCFP fusions are possible.

The promoter region of IscR (P_*iscR*_) was used for *cfp* fusion on plasmid pBE4352::eCFP:eCFP. A DNA fragment with a length of 169 bp was amplified using primers *iscR*_repA and *iscR*_repB. The resulting fragment contains positions −169 to −1with respect to the start codon and includes a predicted IscR interaction site (−119 to −100). Primers (Table S3) generated XbaI/BamHI restriction sites in the corresponding PCR product, which were sub-cloned into the pJET1.2 cloning vector (Thermo Fisher Scientific, Waltham, MA, USA) and after digestion with XbaI/BamHI inserted into pBE4352::eCFP:eCFP. The resulting reporter plasmid pBE::P_*iscR*_::eCFP was used for transformation with *E. coli* S17-1 and subsequently transferred to *R. sphaeroides* by diparental conjugation, yielding the corresponding reporter strain (Table S1).

### UV-visible spectroscopy analysis and fluorescence measurements

The UV-visible spectroscopy analyses of the IscR variants were recorded on a Spectral photometer SPECORD 50 (Analytic Jena, Jena, Germany). Equal amount of proteins were analyzed immediately after protein purification to avoid the oxidation of the Fe–S cluster of the IscR proteins.

For fluorescence measurements a plate reader from the Tecan Infinity M200 series (Tecan, Männedorf, Switzerland) was used. ROS generation was measured using an oxidation-sensitive fluorescent probe, 2,7-dihydrodichlorofluorescein diacetate (DCFH-DA; Thermo Fisher Scientific, Waltham, MA, USA). Exponential cells were incubated with the probe at a final concentration of 10 *μ*mol/L for 30 min. The excitation wavelength was 492 nm, emission wavelength was 525 nm. For *cfp*-based fluorescence measurements the excitation wavelength was 434 nm, the emission wavelength was 480 nm. For both methods the reading mode was top with 0 *μ*sec lag time, 20 *μ*sec integration time, 25 reads, and 0 msec settle time.

### EMSA with dsDNA substrates and IscR protein

For electromobility shifts (EMSAs) the putative regulatory regions (about 200–300 bp) of the respective genes were amplified using the pairs of oligonucleotides shown in Table S3 and were end labeled with [*γ*^32^P]-ATP using T4 polynucleotide kinase (Fermentas). Binding reactions were carried out in a final volume of 15 *μ*L and contained the indicated amount of protein, ∼10 fmol [*γ*^32^P]ATP-labeled DNA probe (10,000 c.p.m.), salmon sperm DNA (1 mg), and 7.5 *μ*L of a binding buffer as described elsewhere (Wu and Outten 2009). The binding reaction was performed at 4°C for 30 min. Samples were loaded on a 6% (w/v) nondenaturing polyacrylamide gel in TBE buffer (22 mmol/L Tris-HCl, 22 mmol/L boric acid, 0.5 mmol/L EDTA (ethylenediaminetetraacetic acid), pH 8.3). Electrophoresis was done at 4°C at 200 V for 4 h.

### RNA isolation and quality assignment

*Rhodobacter sphaeroides* cultures were grown in the presence or absence of iron in triplicate cultures inoculated separately from three independent starter cultures. RNA isolation for quantitative real-time RT-PCR or microarray analysis was performed as previously described (Remes et al. 2014).

### Microarray analysis

Microarray analysis was performed as described before (Peuser et al. 2011). In brief, 2 *μ*g of total RNA of three independent experiments of strains wild-type 2.4.1 and 2.4.1∆*iscR* was chemically labeled with Cy3 and Cy5, respectively. Transcriptome profiles were analyzed on two arrays including six biological replicates. Differentially labeled RNA samples were mixed and competitively hybridized to microarrays. Hybridizations and scanning were performed according to the specifications from Agilent (Böblingen, Germany). Multiarray analysis and normalization according to LOESS were accomplished with the Bioconductor package Limma for R and performed as described elsewhere (Smyth and Speed 2003; Ritchie et al. 2007). On the basis of calculated MA plots, genes were considered reliable if the average signal intensity [A-value: 1/2 log_2_ (Cy3 × Cy5)] was ≥12. Fold changes were calculated using MS Excel (Microsoft Corp. Redmond, WA, USA). The data shown in this study represent the results from two individual microarrays (biological replicates), each containing a pool of three independent experiments for each sample. The microarray data have been deposited in NCBI's Gene Expression Omnibus (Edgar et al. 2002) and are accessible through GEO Series accession number GSE65537 (http://www.ncbi.nlm.nih.gov/geo/query/acc.cgi?acc=GSE65537).

### Quantitative real-time RT-PCR

The One-Step Brilliant III QRT-PCR Master Mix Kit (Agilent) was used for reverse transcription followed by PCR as described in the manufacturer's manual. RT-PCR samples containing 4 ng of total RNA per *μ*L were run in a Rotor-Gene 3000 real-time PCR cycler (Qiagen, Hilden, Germany) for relative quantification of mRNAs in each of the three independent experiments. Primers are listed in Table S3. Crossing points (Cp) with a fluorescence threshold of 0.002 were visualized with the Rotor-Gene software 6.0 (Corbett Research). The relative mRNA levels were normalized to the housekeeping gene *rpoZ* and calculated according to Pfaffl (Pfaffl 2001).

### Homology modeling

The sequence of *R. sphaeroides* IscR was obtained from the Kyoto Encyclopedia of Genes and Genomes (KEGG) (Kanehisa and Goto 2000). IscR has 155 amino acid residues and the accession number is RSP_0443. Comparative modelling was done by searching the protein data bank (PDB) for known protein structures using the sequence of IscR as the query with the program MODELLER (Sali and Blundell 1993). The search was executed using BLASTp, and the results provided three PDB-related potential templates for modelling. The templates are 2Y75, CymR, the global cysteine regulator of *Bacillus subtilis* (Shepard et al. 2011), 4CIC, the transcriptional regulator from *Thermincola potens* (Santos et al. 2014) and 4HF1, HTH-type transcriptional regulator IscR of *E. coli* (Rajagopalan et al. 2013).

## Results

### Organization of *isc* and *suf* genes in a single operon in *R. sphaeroides*

**Figure 1:**
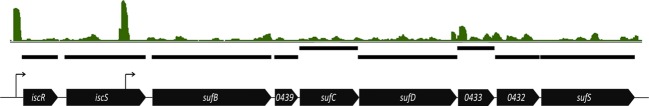
Schematic representation and RNA-seq read coverage of the *isc*-*suf* operon in *Rhodobacter sphaeroides*. Top: Read coverage of the *isc*-*suf* operon in *R. sphaeroides* visualized by the Integrated Genome Browser. Bottom: Schematic representation of the operon. Genes are represented by black boxes and transcriptional start sites are shown by black arrows. The direction of the arrow indicates the direction of transcription.

In contrast to the situation in *E. coli*, the *isc* and *suf* genes that code for Fe–S cluster assembly proteins are in *R. sphaeroides* colocalized on the chromosome. These genes encode a regulator (IscR), two cysteine desulfurases (IscS and SufS), an iron-regulated ABC transporter (SufB), a hypothetical protein (RSP_0439), an ATPase (SufC), an Fe–S cluster assembly protein (SufD), and a putative sulfate transporter (composed of RSP_0433 and RSP_0432) (Fig.1). In a differential RNAseq approach (Sharma et al. 2010) under iron limitation we identified two transcriptional start sites (TSS), one in front of the *iscR* gene, a second within the gene encoding *iscS* (Fig.1) (Remes et al. 2014). This motivated us to test if all genes are part of an operon and are transcribed together. Selected noncoding regions (RSP_0443-0442; RSP_0442-0440; RSP_0440-0439; RSP_0432-0431) were amplified together with upstream and downstream coding regions via RT-PCR. All RT-PCR reactions showed transcripts with the expected size (Fig. S1). To investigate the role of the internal TSS, two sets of primers were used. In the first primer set one primer hybridizes to the 3′ end of *iscS* upstream of the internal TSS, whereas the forward primer of the second primer set hybridizes to the 3′ end of *iscS* downstream of the internal TSS. In both sets the second primer hybridizes to the 5′ end of *sufB*. The amplification products (Fig. S1) revealed the existence of an RNA extending from the 5′ part of *iscS* into *sufB,* strongly implying that transcripts initiating at the TSS in front of *iscR* also comprise the coding regions downstream of *sufB*. We therefore assume that the whole gene cluster is transcribed as one operon, albeit the two promoters may lead to differential expression.

### An *iscR* mutant shows elevated ROS levels and an increased sensitivity to iron limitation

**Figure 2:**
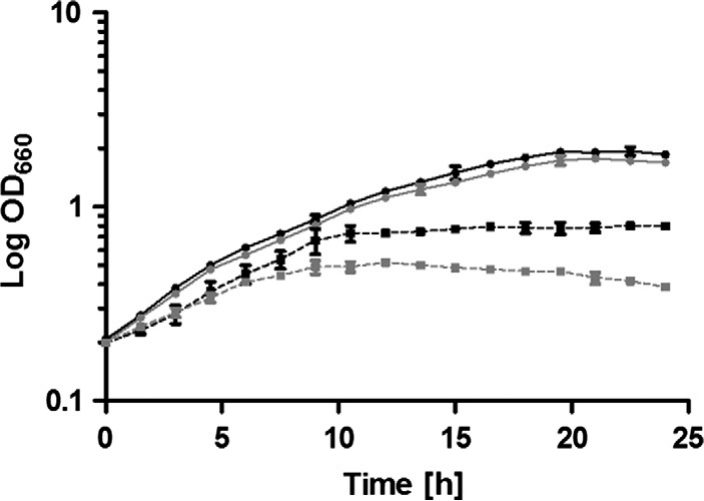
An *iscR* mutant is more sensitive to iron limitation. Growth curves of wild-type *Rhodobacter sphaeroides* (black) and the ∆*iscR* mutant (gray) was performed in iron-replete (continuous line) or iron-limiting (dashed line) conditions. The optical density at 660 nm (OD_660_) was determined over time. The data represent the mean of at least three independent experiments, and the error bars indicate the standard deviation.

**Figure 3:**
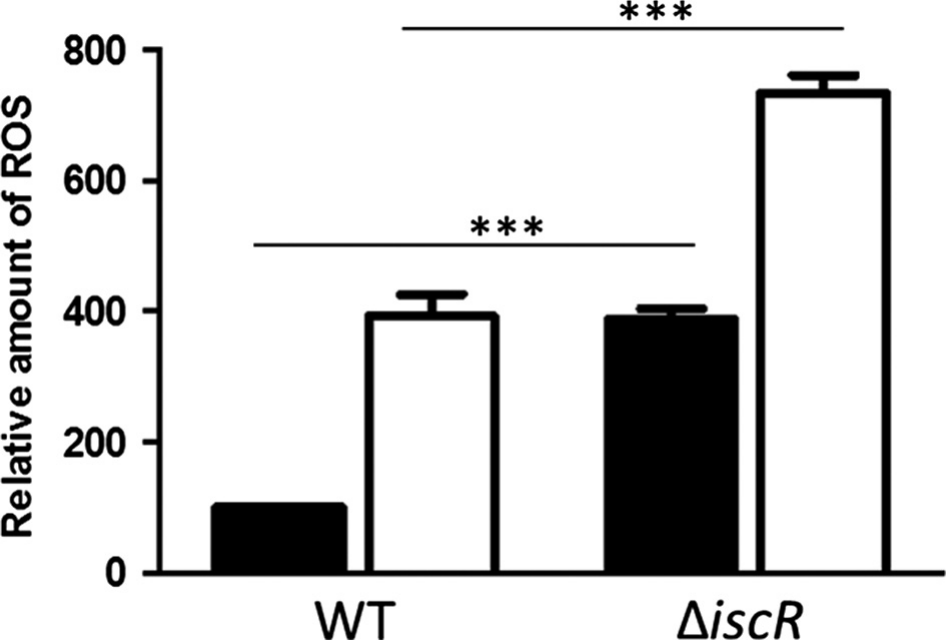
The *iscR* deletion strain shows significantly increased ROS levels. Determination of intracellular levels of ROS in wild-type *Rhodobacter sphaeroides* and the ∆*iscR* mutant strain. Cultures were grown under iron-replete (black) and iron-limiting (white) conditions. ROS generated by the cells were analyzed after reaction with 10 mmol/L 2,7-dihydrodichlorofluorescein diacetate. The fluorescence intensity was normalized to the optical densities of the samples. The resulting values are presented in arbitrary units. The data represent the mean of three independent experiments, and the error bars indicate the standard deviation. A *P*-value was computed using the student's *t* test. ***Significant at *P* ≤ 0.001.

To elucidate the role of the predicted regulator encoded by the first gene of the operon, an *iscR* deletion strain was constructed as described in Materials and Methods. The growth of the Δ*iscR* mutant was similar to that of the wild-type in iron-replete conditions, whereas growth of the mutant was more severely impeded than that of the wild-type in iron-limiting conditions (Fig.2). To investigate the impact of oxidative stress on this growth behavior, ROS levels were monitored in both strains. As previously described, exposure to iron starvation cause a strongly increased ROS accumulation in the wild-type cells (Peuser et al. 2011; Remes et al. 2014). An *iscR* mutant showed in comparison to the wild-type significantly increased ROS production irrespective of iron availability (Fig.3).

### IscR regulates genes involved in iron and sulfur metabolism

A high-density oligonucleotide microarray was applied for comparing gene expression in strain ∆*iscR* and its parental wild-type strain. A total of 110 protein coding genes are differently expressed (log_2_ fold change ≥0.6 or ≤ −0.6) in the absence of *iscR* (Table S4). Table1 gives an overview of differently expressed genes involved in iron homeostasis as well as genes preceded by an Iron-Rhodo-box motif. The *iscR* deletion strain carries an antibiotic resistance cassette within the *iscR* gene, which also abolishes expression of *iscS*, while three genes (*sufCD*, RSP_0439) of the *isc*-*suf* operon were strongly expressed. Indeed, besides the promoter upstream of *iscR* an additional promoter element was identified by an RNAseq approach upstream of *sufB* (Fig.1). Thus, transcription of the *sufBCD* genes in the *iscR* mutant seems to originate from this additional, *iscR*-independent promoter. Nevertheless, as mentioned above, we proved the existence of an RNA extending from the 5′ part of *iscS* into *sufB* (Fig. S1). According to this, the *suf* transcripts are at least partly initiated at the *iscR* promoter in the wild-type situation.

**Table 1:** Selection of IscR-responsive genes in *Rhodobacter sphaeroides* as determined by microarray analysis

RSP no.	Gene	log_2_ FC	Description
**RSP_0437**	*sufC*	0.68	Suf C, ATPase
**RSP_0439**		0.67	Hypothetical protein
**RSP_0440**	*sufB*	0.67	Putative SufB
**RSP_0442**	*iscS*	−1.00	Putative aminotransferase
**RSP_0443**	*iscR*	−1.15	Rrf2 family transcriptional regulator
**RSP_0920**	*exbB*	1.98	Biopolymer transport protein, ExbB
**RSP_0921**	*exbD*	1.59	Biopolymer transport protein, ExbD
**RSP_0922**	*tonB*	1.59	Putative TonB protein
RSP_1438		0.62	ABC Fe hydroxamate (ferrichrome) transporter, fused inner membrane subunits
RSP_1440		0.93	TonB dependent, hydroxamate-type ferrisiderophore, outer membrane receptor
**RSP_1543**		0.72	Hypothetical protein
**RSP_1544**		0.92	Hypothetical signal peptide protein
**RSP_1545**		(0.59)	Probable thiol oxidoreductase with 2 cytochrome c heme-binding sites
**RSP_1546**	*bfr*	1.50	Bacterioferritin
**RSP_1547**	*bfd*	2.30	Probable bacterioferritin-associated ferredoxin
**RSP_1548**		2.53	Putative iron-regulated protein
RSP_1818	*feoB*	0.86	Fe2 transport system protein B
RSP_1819	*feoA1*	0.87	Ferrous iron transport protein A
RSP_1949		0.65	FeS assembly SUF system protein
**RSP_2913**	*afuA*	1.93	ABC Fe siderophore transporter, periplasmic substrate-binding protein
RSP_3076		0.75	Hypothetical protein
RSP_3077		0.95	Hypothetical protein
RSP_3078		1.01	Hypothetical protein
RSP_3079		0.66	ABC Fe siderophore transporter, periplasmic substrate-binding protein
RSP_3223		0.85	TonB-dependent receptor precursor
**RSP_3416**		(0.07)	ABC Fesiderophore transporter, periplasmic-binding protein
**RSP_3417**		(0.00)	TonB-dependent outer membrane ferrichrome-iron receptor
RSP_3678		0.77	Siderophore-interacting protein
**RSP_4275**	*fecI*	(0.32)	sigma24, FecI
**RSP_6006**		2.53	Hypothetical protein
RSP_6020	*feoA2*	1.15	Ferrous iron transport protein A

Significant expression changes (log_2_ fold change ≥0.6 or ≤0.6) of selected genes (2.4.1∆*iscR* vs. wild-type) are shown. Genes with RSP numbers in boldface type are located in operons preceded by an Iron-Rhodo-box motif. Numbers in parentheses did not pass our selection criteria (log_2_ FC ≥0.6 or ≤ −0.6).

Many genes with predicted functions in ferric/ferrous iron uptake or iron storage showed a higher expression in the mutant strain, including *exbBD-tonB*, RSP_1438-1440 encoding a ferrichrome transporter, *bfd*-*bfr* encoding a bacterioferritin, *irpA* encoding an iron-regulated protein, *feoAB* encoding a ferrous transport system, genes encoding Fe^3+^-siderophore transporters (*afuA*, RSP_3079, RSP_3678) and *hemP* encoding an iron uptake protein. Furthermore, several genes encoding proteins involved in adaptation to cold stress (RSP_1952, RSP_3260/21), oxidative stress (*rpoE,* RSP_1091, *phrA*), glycolysis (RSP_2968), pyruvate decarboxylation (*pdhAB*), flagellum biosynthesis (*fli*, *flg*) and chemotaxis (*che*) had significantly lower expression levels in the mutant compared to the wild-type (Table S4).

To validate the microarray data real-time RT-PCR was used for the quantification of mRNAs transcribed from several selected genes. In addition to genes involved in iron homeostasis one gene of the flagellum biosynthesis (*fliS*), the ABC zinc transporter gene *znuA*, a gene for bacteriochlorophyll synthesis (*bchL*), and for a structural protein of the reaction center (*pufL*) were selected for validation. Increase or decrease of expression levels as revealed by microarray analysis were confirmed by real-time RT-PCR (Fig. S2). Nevertheless for some genes, the extent of change varied between the two approaches and was mostly more pronounced in the real-time RT-PCR data set. As a consequence some genes showed a clear difference in expression between the two strains only in the RT-PCR analysis (e.g. RSP_3416 and RSP_3417).

### *Rhodobacter sphaeroides* IscR exhibits a new type of Fe–S coordination

**Figure 4:**
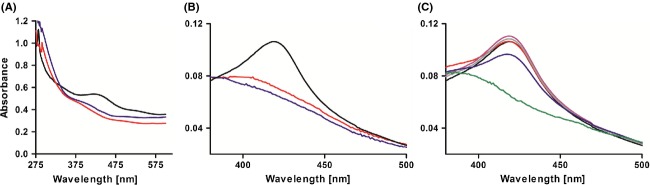
UV-visible absorption spectra of various IscR samples in native state. (A) UV-vis spectra of purified IscR (∼60 *μ*mol/L). After Ni-NTA purification, His-tagged IscR was first oxidized (red), then reconstituted in strictly anaerobic conditions (black), and finally oxidized again (Blue). (B) UV-Vis spectra of His-tagged IscR (∼10 *μ*mol/L) purified under reduced conditions (black), iron-limiting conditions (red), or treated with 1 mmol/L H_2_O_2_ (blue), respectively. (C) UV-Vis spectra of His-tagged IscR (∼10 *μ*mol/L) (black) and its single-site mutants H93A (red), H121A (gray), H127A (purple), C142A (green), P143A (yellow), and the triple-site mutant H93A/H121A/H127A (blue), respectively.

Apo-IscR typically ligates an Fe–S cluster in its C-terminal part by three Cys residues and one histidine residue. This motif is conserved in many proteobacteria (Fleischhacker et al. 2012), but only a single Cys residue is present in the C-terminal part of *Rhodobacteraceae* IscR at a position not matching the Fe–S coordinating Cys residues of other IscR proteins (Fig. S3). We used the oxidized apo-form of IscR for chemical reconstitution under strictly anaerobic conditions, yielding a brown protein solution. The reconstituted protein showed the typical absorption shoulder at 410–420 nm, which is characteristic for iron-sulfur proteins (Fig.4A) (Kulzer et al. 1998; Zeng et al. 2008). This absorption pattern disappeared after cluster reduction in aerobic conditions (Fig.4A).

For further analysis, we measured absorption spectra of reduced IscR, which showed a similar absorption pattern with a peak at 420 nm (Fig.4B). By contrast, IscR grown and purified under iron-limiting conditions showed nearly no absorption at 420 nm relative to the control, indicating a strongly reduced amount of Fe–S clusters ligated to the protein (Fig.4B). To determine the effect of ROS on Fe–S cluster integrity, purified His-tagged IscR was incubated with H_2_O_2_ (1 mmol/L) for 2 min prior to UV-visible spectroscopy. The results showed a total loss in IscR absorbance at 420 nm (Fig.4B), suggesting that in the presence of oxidative stress, the Fe–S clusters ligated to IscR were targets for H_2_O_2_ -mediated oxidation that resulted in destabilization of Fe–S bound to the protein.

Since other IscR proteins coordinate the Fe–S cluster via 3 Cys and one His residue we replaced the single Cys and the three His residues in the C-terminal part of *R. sphaeroides* IscR. His-tagged IscR and IscR variants carrying these amino acid substitutions (H93A, H121A, H127A, and C142A) were purified and equal amounts of the proteins were then subjected to UV-visible spectroscopy. The IscR variant C142A had nearly no absorption at 420 nm indicating that binding of Fe–S cluster to this protein is severely impaired (Fig.4C). In contrast, the three mutated IscR variants H93A, H121A and H127A and also the triple mutant H93A/H121A/H127A showed the typical IscR absorption at 420 nm.

Furthermore, *R. sphaeroides* IscR harbors the so-called “heme regulatory motif” (HRM) in its C-terminal region. HRMs are heme-binding sequences that are found in proteins involved in many aspects of heme and iron metabolism. The consensus sequence comprises a stretch of residues where only a Cys-Pro dipeptide is absolutely conserved and in most cases flanked by hydrophobic amino acids (Lathrop and Timko 1993; Zhang and Guarente 1995). Thus, the Pro_141_-Cys-Pro-Ala-Val_145_ sequence reflects a typical HRM found in proteins that bind heme. To exclude that the observed absorption peak at 420 nm is due to a heme bound to the HRM rather than to an Fe–S cluster, we mutated the essential amino acids in the conserved HRM. While a total loss in absorption was observed for the IscR variant C142A, IscR variant P143A shows the same absorption at 420 nm as the wild-type (Fig.4C).

### IscR requires an Fe–S cluster to repress target genes with an Iron-Rhodo-box motif

**Figure 5:**
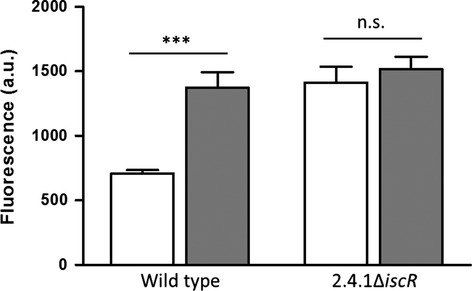
IscR negatively regulates expression of its own promoter (P_*iscR*_). Relative fluorescence was measured from the wild-type strain and deletion strain ∆*iscR*, both containing the eCFP:P_*iscR*_ reporter fusions on plasmid pBE. Strains were grown in iron-replete (white bars) or iron-limiting (gray bars) conditions. The data represent the average activity of three independently isolated strains. A *P*-value was computed using the student's *t* test. Variations were considered statistically significant when the *P*-value was ≤0.05. ***Significant at *P* ≤ 0.001.

In the *Rhodobacteraceae*, a highly conserved 19-bp palindromic signal, termed Iron-Rhodo-box, is located in the regulatory regions upstream of genes encoding iron uptake and storage proteins, and was predicted for an unknown regulator or for IscR (Rodionov et al. 2006). Expression analyses revealed that most of these genes are induced in response to iron starvation in wild-type cells (Peuser et al. 2011; Remes et al. 2014), and all genes showed higher expression in iron replete conditions in the background of ∆*iscR* (Fig. S4A). However, deletion of *iscR* resulted in an abolished expression of the downstream gene *iscS*. In the complemented strain ∆*iscR*_pBBR*iscR*, the expression of the target genes reached wild-type-like levels (Fig. S4B), while the transcript level of *iscS* is unchanged in comparison to the *iscR* mutant (data not shown). Thus, the repression of its target genes is solely IscR dependent. However, no difference or even lower expression levels were observed in the mutant compared to the parental wild-type strain in iron-limiting conditions (Fig. S4A). To further investigate a potential Fe–S cluster requirement for the repressor function of IscR to its own promoter (P_*iscR*_), we measured the activity of P_*iscR*_ in both, iron-deplete and iron-replete conditions. For this approach, the target promoter region P_*iscR*_ was transcriptionally fused to the *cfp* gene on plasmid pBE4352: eCFP: eCFP and transformed in both, wild-type and *iscR* deletion strain. In the presence of iron, deletion of *iscR* results in a significant induction of P_*iscR*_ activity (Fig.5). However, under iron-limiting conditions, IscR exhibited a severe defect in P_*iscR*_ repression (Fig.5). Thus, IscR negatively regulates its own transcription only in the presence of iron.

To study the DNA-binding function of IscR with predicted *iscR*-controlled promoters, we chose the *iscR* and *hemP* promoter regions for binding assays. His-tagged IscR was purified after heterologous overexpression and radiolabeled DNA probes containing the predicted binding sites were co-incubated and separated on a nondenaturing polyacrylamide gel. With increasing IscR concentrations, formation of a retarded DNA protein complex was observed for both fragments (Fig. S5, lane 2–5). Addition of an up to 100-fold molar excess of an unlabeled nonspecific probe containing the regulatory region of *znuA* did not interfere with the complex formation, confirming specificity of binding (Fig. S5, lane 6–7). The *znuA* gene is IscR-dependently regulated (Table S4) but does not carry the Iron-Rhodo-box motif. However, almost a complete loss of the retardation was observed when 10-fold molar excess of the specific unlabeled DNA probe was used to compete out the labeled probe (Fig. S5, lane 8–9). This result indicates that genes preceded by the Iron-Rhodo-box motifs are under direct control of IscR.

**Figure 6:**
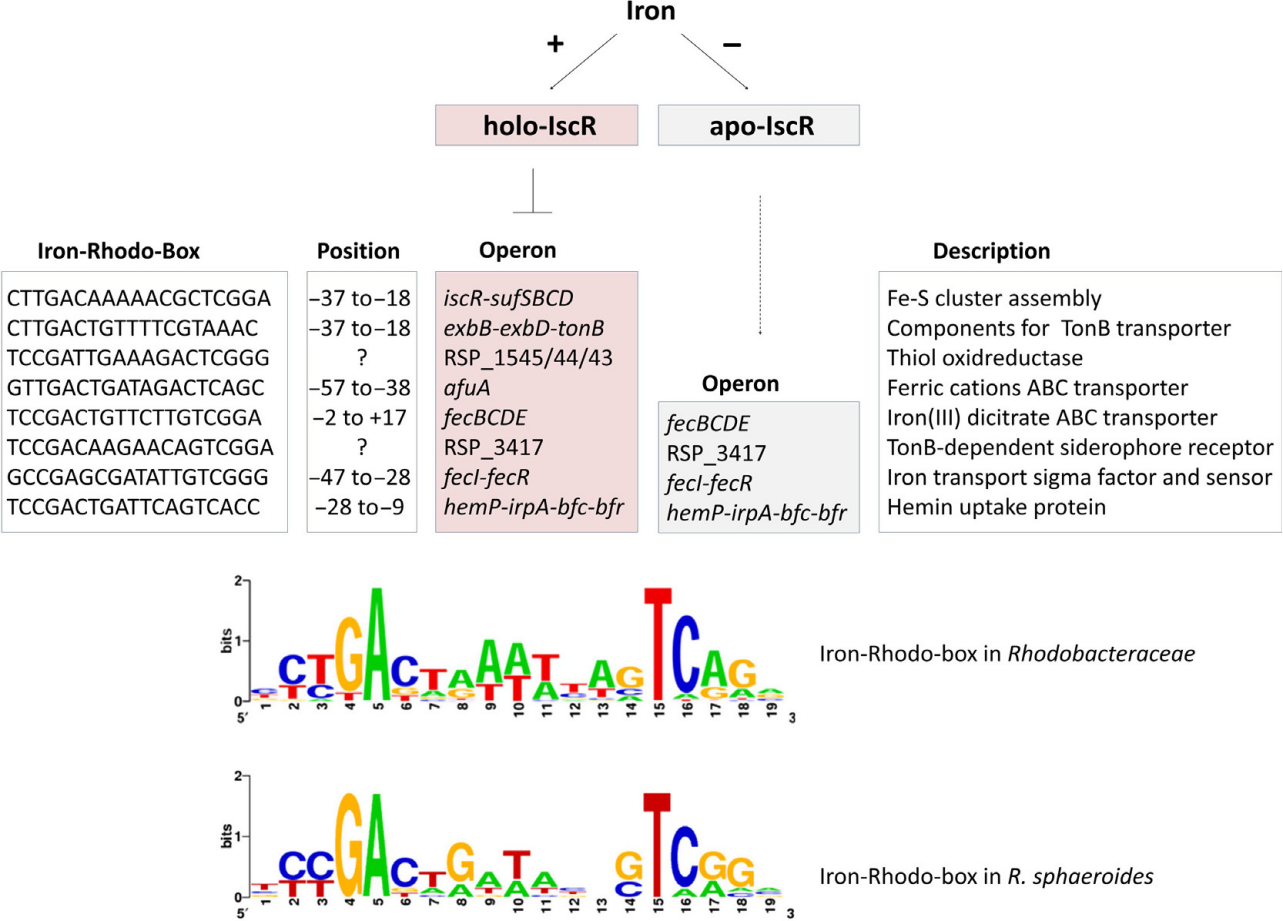
Model of the IscR regulon in *Rhodobacter sphaeroides*. Fe–S cluster-containing IscR inhibits transcription of all genes preceded by an Iron-Rhodo-box motif, while apo-IscR activates transcription of some of them. The position relative to the transcriptional start site as determined by differential RNAseq (Remes et al. 2014) is shown. A consensus logo of the Iron-Rhodo-box motif for the *Rhodobacteraceae* (Rodionov et al. 2006) and for *R. sphaeroides* was drawn, using the WebLogo package version 2.8.2 (http://weblogo.berkeley.edu).

Figure6 illustrates the effect of IscR on the *R. sphaeroides* genes with predicted Iron-Rhodo-boxes. Based on previous dRNAseq analyses (Remes et al. 2014), we also show the position of the Iron-Rhodo-box motif in relation to the TSS. RSP_3417 was only very weakly expressed under the chosen conditions and no TSS could be identified. No TSS could be identified directly upstream of RSP_1545. The dRNAseq data strongly suggest that RSP_1545-1543 are cotranscribed together with *hemP-irpA-bfd-bfr*. This suggests one IscR-binding site directly in the *hemP* promoter and an additional binding site within the operon. All other IscR-binding sites including that upstream of *hemP* completely or partially overlap the −35/−10 region or are positioned very close to it as in the case of the *afuA* promoter. The consensus sequence of these motifs matches well to the consensus sequence for the Iron-Rhodo-box motif of *Rhodobacteraceae* as published by Rodionov et al. (2006).

## Discussion

*E. coli* IscR acts as a sensor of the cellular demands for Fe–S cluster biogenesis by binding an Fe–S cluster with (Cys)_3_(His)_1_ (Fleischhacker et al. 2012). IscR thereby acts as global regulator involved in a homeostatic mechanism controlling Fe–S cluster biogenesis (Giel et al. 2006; Lee et al. 2008; Wu and Outten 2009; Giel et al. 2013). In *E. coli*, holo-IscR directly represses the Isc Fe–S biogenesis pathway, while as an apo-protein, it activates an alternative Fe–S biogenesis pathway, encoded by the *suf* genes (Schwartz et al. 2001; Giel et al. 2006; Yeo et al. 2006; Lee et al. 2008). An important question was therefore, whether the unusual *R. sphaeroides* IscR serves a similar function as its *E. coli* homolog and is able to ligate an Fe–S cluster.

In contrast to all other IscR proteins from proteobacteria, IscR proteins from *Rhodobacteraceae* lack the three conserved Cys residues, which are essential for Fe–S cluster ligation (Rodionov et al. 2006; Yeo et al. 2006; Nesbit et al. 2009). A phylogenetic tree revealed that IscR proteins from *Rhodobacteraceae* form a distinct cluster within the group of alpha proteobacteria (Rodionov et al. 2006) (Fig. S6). There is only a single-Cys residue present in the C-terminal region and it was therefore proposed that *Rhodobacteraceae* IscR is unlikely to ligate an Fe–S cluster (Rodionov et al. 2006). However, our data confirm that *R. sphaeroides* IscR contains an Fe–S cluster and that the single Cys residue is essential for this ligation (Fig.4).

A homology model based on the comparative modelling method from known PDB-related three-dimensional structures shows that this Cys residue is surface-exposed (Fig. S7). It is therefore conceivable that apo-IscR coordinates one Fe–S cluster by Cys residues of distinct IscR protomers. Similar results were observed for *Aquifex aeolicus* IscU and *Thermosynechococcus elongates* IscA, which coordinate one Fe–S cluster by two or three conformationally distinct protomers, respectively (Morimoto et al. 2006; Shimomura et al. 2008). Although, both proteins contain the three-conserved Cys residues. To analyze if *R. sphaeroides* IscR forms an oligomeric complex, the purified protein was subjected to gel filtration on a Superdex 200 16/60 column. The elution profile indicated that IscR even under aerobic conditions forms dimers and tetramers (data not shown). In Rieske proteins, the Fe–S cluster is ligated by an unusual (Cys)_2_(His)_2_, but no His residues are involved in *R. sphaeroides* IscR Fe–S cluster binding (Fig.4C). However, it is conceivable that the cluster is coordinated by three additional noncysteinyl ligands. Further biochemical analysis and structural characterization is required to establish a clear picture of the Fe–S cluster ligation and to identify the precise composition of Fe–S clusters bound to IscR.

In contrast to the situation in *E. coli*, the *isc* and *suf* genes in *Rhodobacteraceae* are co-localized on the chromosome and in *R. sphaeroides* transcribed as one operon (Fig. S1). Several genes of the operon are highly expressed under various oxidative stresses or iron-limiting conditions to compensate the decreased Fe–S availability (Zeller et al. 2007; Peuser et al. 2011; Berghoff et al. 2013; Remes et al. 2014). Regulation of the *R. sphaeroides isc*-*suf* operon by oxidative stress is under control of the global regulator OxyR, which senses H_2_O_2_ (Zeller et al. 2007; Remes et al. 2014). An *oxyR* mutant showed increased ROS production and impaired growth under iron limitation (Remes et al. 2014). The same phenotype was observed for the *iscR* deletion strain of *R. sphaeroides* (Figs.2, 3) and of *Pseudomonas aeruginosa* (Romsang et al. 2014). It is likely that the lack of IscR prevents activation of iron uptake genes, leading to lower intracellular iron concentrations than in the wild-type, and consequently to an impaired growth behavior.

IscR has recently emerged as a pleiotropic regulator that influences the expression of ∼40 genes in *E. coli* or 67 genes in *Vibrio vulnificus* (Giel et al. 2006; Lim and Choi 2014). Our transcriptome study identified an *R. sphaeroides* IscR regulon comprising ∼110 protein coding genes. Deletion of *iscR* also abolished expression of *iscS*. Although this had no influence on genes with predicted Iron-Rhodo-boxes (Fig. S4B), we cannot completely rule out that some genes of the regulon are affected by the deletion of *iscS*. The IscR regulons of the three organisms *E. coli*, *V. vulnificus,* and *R. sphaeroides* share important functions, including iron homeostasis, motility or oxidative stress response. For most of the *R. sphaeroides* regulated genes, a similar expression pattern was previously observed for the wild-type strain in response to iron limitation in oxic conditions, potentially due to elevated ROS levels (Peuser et al. 2011; Remes et al. 2014). In agreement with this, of the 110 IscR-dependent genes 33 are affected by iron levels, 28 by hydrogen peroxide, and 44 by singlet oxygen stress (Zeller et al. 2005; Peuser et al. 2011; Berghoff et al. 2013). The increased ROS levels in strain ∆*iscR* compared to the wild-type in iron-replete and iron-deplete conditions (Fig.3) support an indirect regulation for those genes in response to oxidative stress.

Oxidative stress and iron limitation result in a decrease of holo-IscR (Fig.4B), but in an increased *iscR* mRNA level (Zeller et al. 2005; Peuser et al. 2011; Remes et al. 2014), Since the repressor function of IscR in *R. sphaeroides* was no longer present under iron-limiting conditions (Figs.5, S4A), the cluster coordination is a necessary feature for IscR to repress its target promoters. In *E. coli* transcriptional regulators, such as IscR and SoxR, bind their DNA targets independently of an Fe–S cluster, while transcription of target genes is cofactor dependent (Hidalgo et al. 1998; Nesbit et al. 2009; Rajagopalan et al. 2013). Binding of *R. sphaeroides* IscR to the *iscR* and *hemP* promoter region did also not require the Fe–S cluster, but IscR only represses both genes in its holo-form. However, IscR also activates some genes as apo-protein (Fig. S4A), maximizing the cellular capacity for iron uptake, Fe–S cluster assembly and maintenance.

Since no IscU scaffold protein is present in *R. sphaeroides*, it was proposed that a complex of SufBCD could function as both, scaffold protein, and transporter for Fe–S cluster to apo-IscR (Wollers et al. 2010; Vinella et al. 2013). Therefore, the SufBCD proteins have to be able to distinguish between IscR and other apo-protein targets. While the details of this putative target specificity remain disputable, the ligation scheme with only one Cys may differentiate IscR from other apo-proteins. Due to this ligation scheme *R. sphaeroides* IscR exhibits probably a decreased affinity for Fe–S cluster and is only able to bind the clusters if no other proteins require them. We propose that sufficient amounts of Fe–S cluster are sensed by forming holo-IscR, while in turn P_*iscR*_ gets repressed by a negative feedback loop to keep appropriate levels of cellular Fe–S cluster formation and delivery.

## Supporting Information

**Figure S1.** The *Rhodobacter sphaeroides isc*-*suf* operon is cotranscribed as determined by RT-PCR. Predicted RT-PCR products are represented by lines under the genes and predicted RT-PCR product sizes are shown in parentheses. RT-PCR was performed with total RNA in the presence (+) or absence (−) of reverse transcriptase or with genomic DNA (G).

**Figure S2.** Validation of microarray data by real-time RT-PCR. Quantified log_2_ fold changes of iron-responsive genes in 2.4.1Δ*iscR* compared to the wild-type was determined by real-time RT-PCR. Values are normalized to that of the housekeeping gene *rpoZ*. The data presented are the means of at least three experiments, and the standard deviations of the means are indicated (error bars). Numbers in parentheses show the fold change of the respective genes as determined by microarray analysis.

**Figure S3.** Similarity of *Rhodobacter sphaeroides* IscR to *Escherichia coli* IscR. Amino acid sequence alignment of the two proteins generated with “align” showed 43% identical residues (*) and 23% similar residues (:). Solid lines denote the helix-turn-helix DNA-binding domains; the three Cys residues (C92, C98, and C104) of *E. coli* IscR that coordinate the Fe–S cluster are framed.

**Figure S4.** Functional analysis of IscR repressor activity via real-time RT PCR. (A) Relative gene expression in strain 2.4.1Δ*iscR* in comparison to that of the wild-type under iron-replete conditions (black) or under iron limitation (gray). (**B**) Relative gene expression of the mutant 2.4.1Δ*iscR* (black) or the complemented mutant Δ*iscR*_pBBR*iscR* (white) in comparison to the wild-type under iron-replete conditions. Values are normalized to that of the housekeeping gene *rpoZ*. The data represent the mean of at least three independent experiments. A *P*-value was computed using the student's *t* test. Variations were considered statistically significant when the *P*-value was ≤0.05. *Significant at *P* ≤ 0.05; **significant at *P* ≤ 0.01; ***significant at *P* ≤ 0.001.

**Figure S5.** Binding of purified IscR to the promoter regions of *iscR* (A) and *hemP* (B) as determined by EMSAs. All reactions contain the same amount of ^32^P end-labeled DNA fragment (∼5 fmol/lane) comprising the promoter sequence, including the Iron-Rhodo-box motif. Lane 1 contains no IscR; lanes 2–5 contain increasing amounts of IscR (100–500 ng); lanes 6–9 contain 500 ng IscR; lanes 6 and 7 contain excess amounts of an unlabeled nonspecific probe containing the regulatory region of *znuA*. Lanes 8–9 contain excess amounts of the respective unlabeled DNA probe as competitor.

**Figure S6.** (A) Phylogenetic Tree of IscR from proteobacteria (modified from Rodionov et al. 2006; Fig. 3B). The genome abbreviations are listed in (**B**).

**Figure S7.** Homology model of the IscR monomer calculated using the MODELLER program. The cartoon representation in blue of the IscR protein shows a helix-turn-helix fold. The surface accessible residue Cys-142 is represented as a stick model in magenta.

**Table S1.** *Rhodobacter sphaeroides* and *Escherichia coli* strains used in this study. **Table S2.** Plasmids used in this study. **Table S3.** Oligodeoxynucleotide used in this study.

**Table S4.** Gene expression changes in 2.4.1Δ*iscR* as determined by microarray analysis.
