# Supplementary figures and images for: IscR of Rhodobacter sphaeroides functions as repressor of genes for iron-sulfur metabolism and represents a new type of iron-sulfur-binding protein
Source: Microbiologyopen. 2015 Aug 1;4(5):790–802. doi: 10.1002/mbo3.279 (PMC4618611; doi:10.1002/mbo3.279)

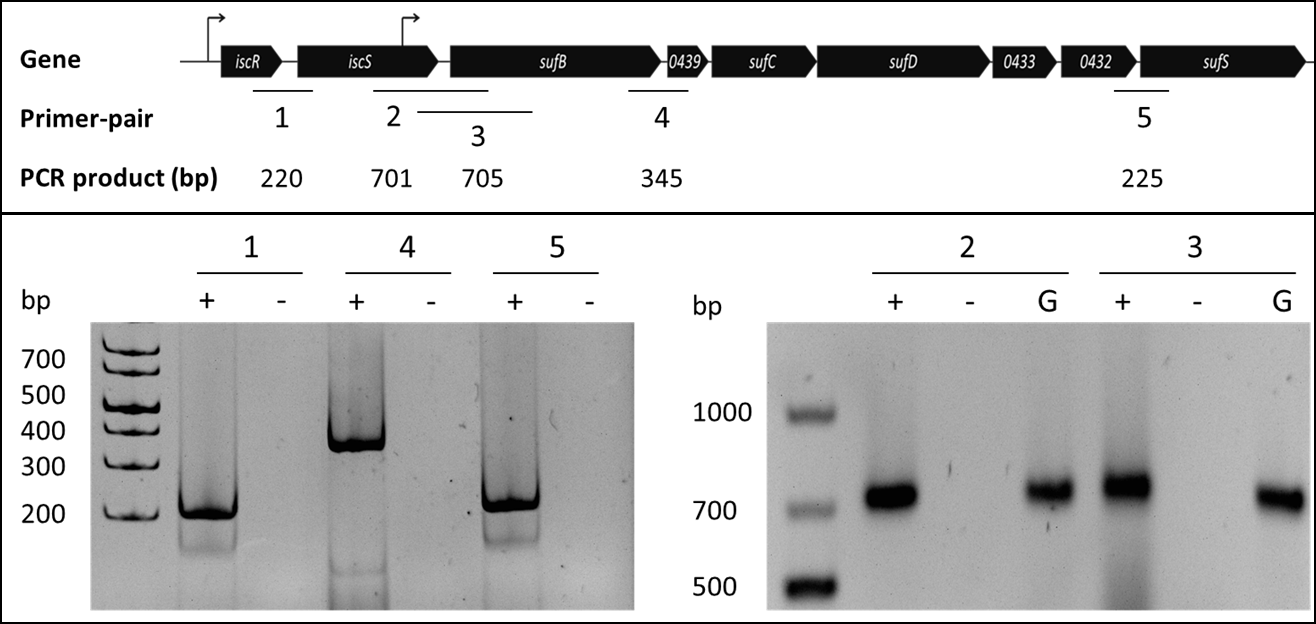

Supplement: Supplementary file 1 — Figure S1. The Rhodobacter sphaeroides isc-suf operon is cotranscribed as determined by RT-PCR. Predicted RT-PCR products are represented by lines under the genes and predicted RT-PCR product sizes are shown in parentheses. RT-PCR was performed with total RNA in the presence (+) or absence (−) of reverse transcriptase or with genomic DNA (G). [file mbo30004-0790-sd1.tif]

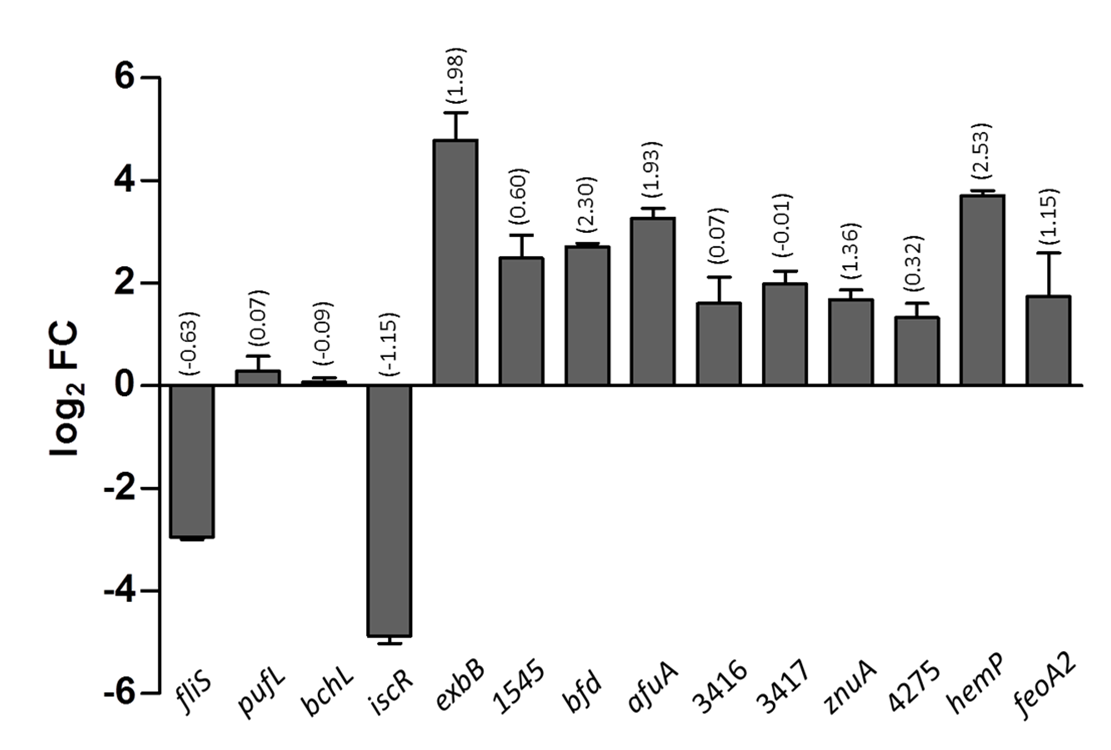

Supplement: Supplementary file 2 — Figure S2. Validation of microarray data by real-time RT-PCR. Quantified log2 fold changes of iron-responsive genes in 2.4.1ΔiscR compared to the wild-type was determined by real-time RT-PCR. Values are normalized to that of the housekeeping gene rpoZ. The data presented are the means of at least three experiments, and the standard deviations of the means are indicated (error bars). Numbers in parentheses show the fold change of the respective genes as determined by microarray analysis. [file mbo30004-0790-sd2.tif]

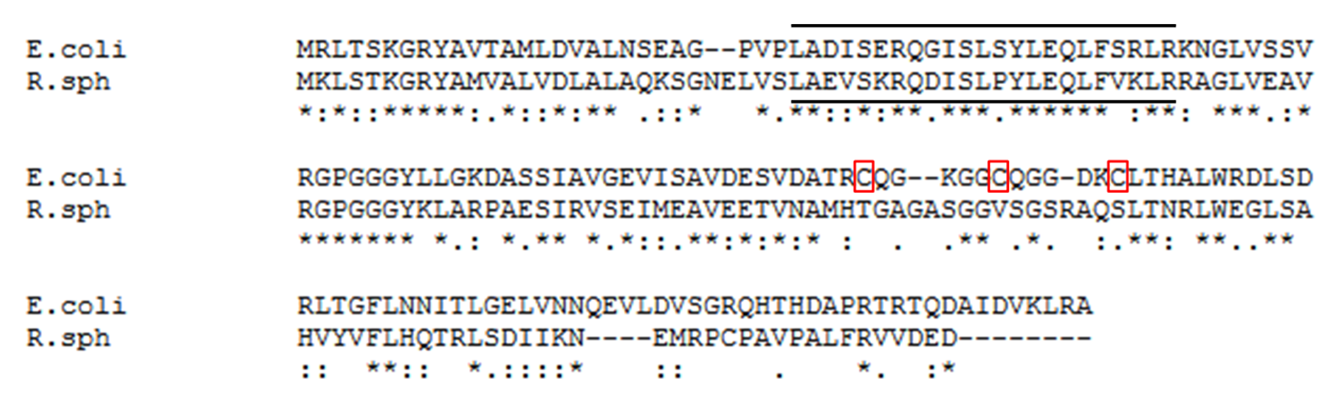

Supplement: Supplementary file 3 — Figure S3. Similarity of Rhodobacter sphaeroides IscR to Escherichia coli IscR. Amino acid sequence alignment of the two proteins generated with “align” showed 43% identical residues (*) and 23% similar residues (:). Solid lines denote the helix-turn-helix DNA-binding domains; the three Cys residues (C92, C98, and C104) of E. coli IscR that coordinate the Fe–S cluster are framed. [file mbo30004-0790-sd3.tif]

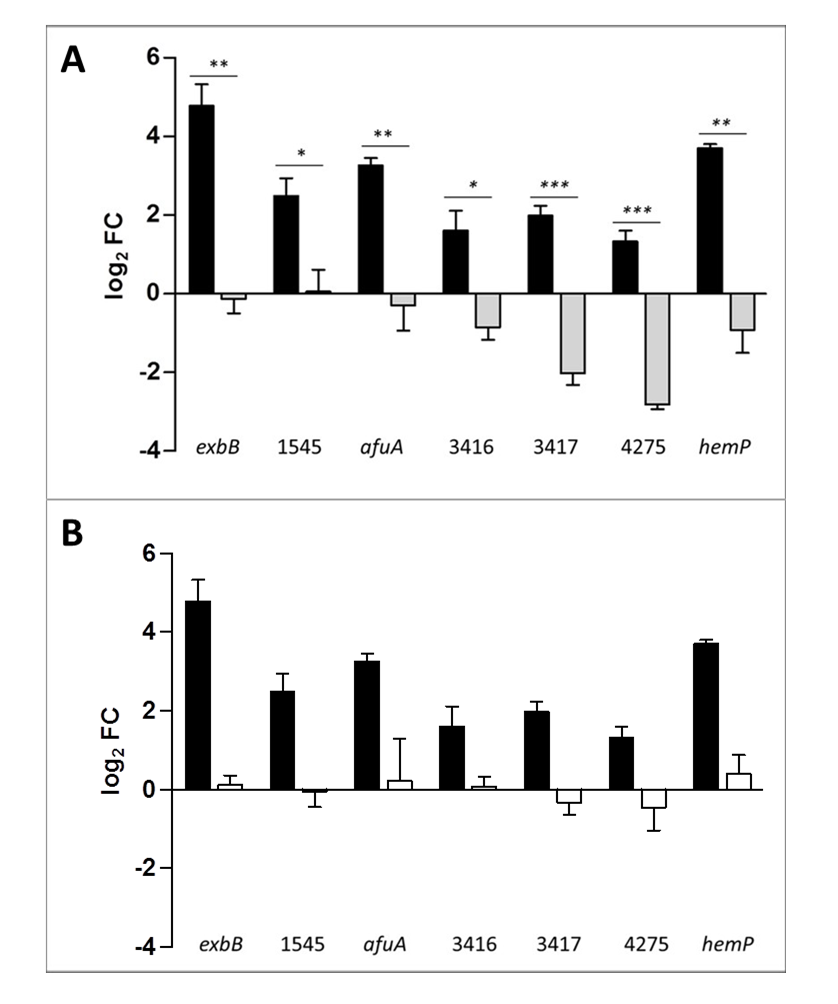

Supplement: Supplementary file 4 — Figure S4. Functional analysis of IscR repressor activity via real-time RT PCR. (A) Relative gene expression in strain 2.4.1ΔiscR in comparison to that of the wild-type under iron-replete conditions (black) or under iron limitation (gray). (B) Relative gene expression of the mutant 2.4.1ΔiscR (black) or the complemented mutant ΔiscR_pBBRiscR (white) in comparison to the wild-type under iron-replete conditions. Values are normalized to that of the housekeeping gene rpoZ. The data represent the mean of at least three independent experiments. A P-value was computed using the student's t test. Variations were considered statistically significant when the P-value was ≤0.05. *Significant at P ≤ 0.05; **significant at P ≤ 0.01; ***significant at P ≤ 0.001. [file mbo30004-0790-sd4.tif]

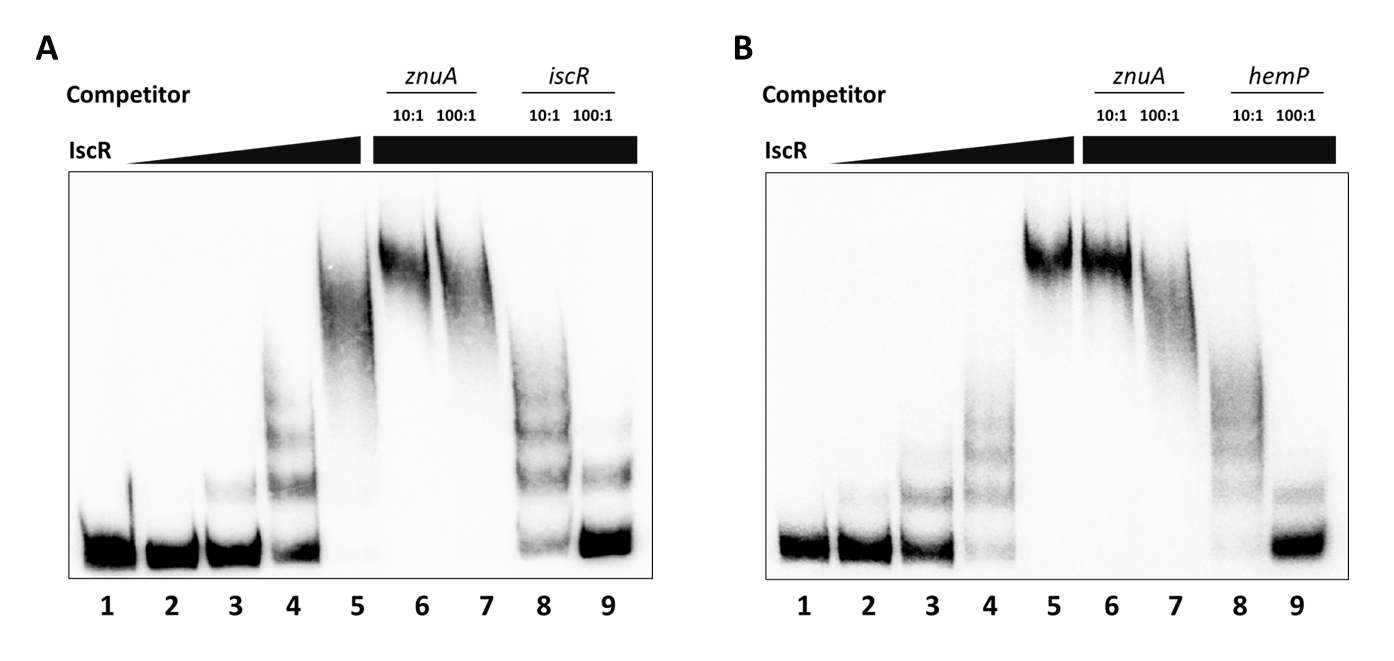

Supplement: Supplementary file 5 — Figure S5. Binding of purified IscR to the promoter regions of iscR (A) and hemP (B) as determined by EMSAs. All reactions contain the same amount of 32P end-labeled DNA fragment (∼5 fmol/lane) comprising the promoter sequence, including the Iron-Rhodo-box motif. Lane 1 contains no IscR; lanes 2–5 contain increasing amounts of IscR (100–500 ng); lanes 6–9 contain 500 ng IscR; lanes 6 and 7 contain excess amounts of an unlabeled nonspecific probe containing the regulatory region of znuA. Lanes 8–9 contain excess amounts of the respective unlabeled DNA probe as competitor. [file mbo30004-0790-sd5.tif]

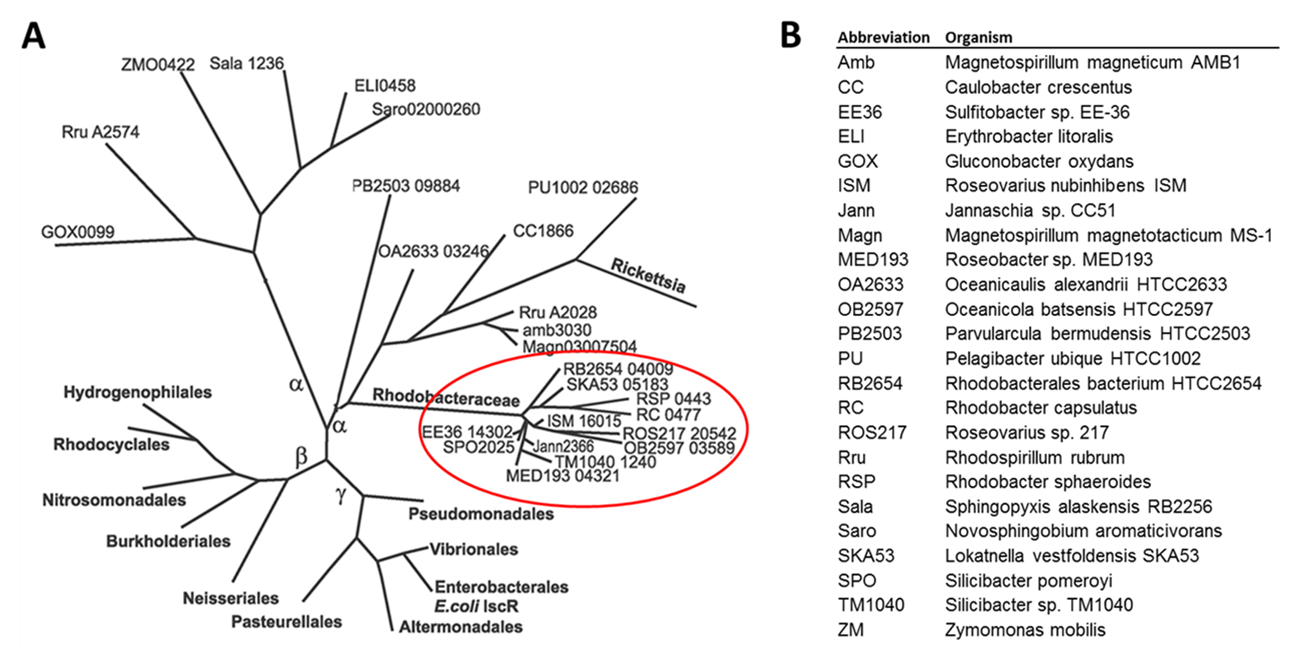

Supplement: Supplementary file 6 — Figure S6. (A) Phylogenetic Tree of IscR from proteobacteria (modified from Rodionov et al. 2006; Fig. 3B). The genome abbreviations are listed in (B). [file mbo30004-0790-sd6.tif]

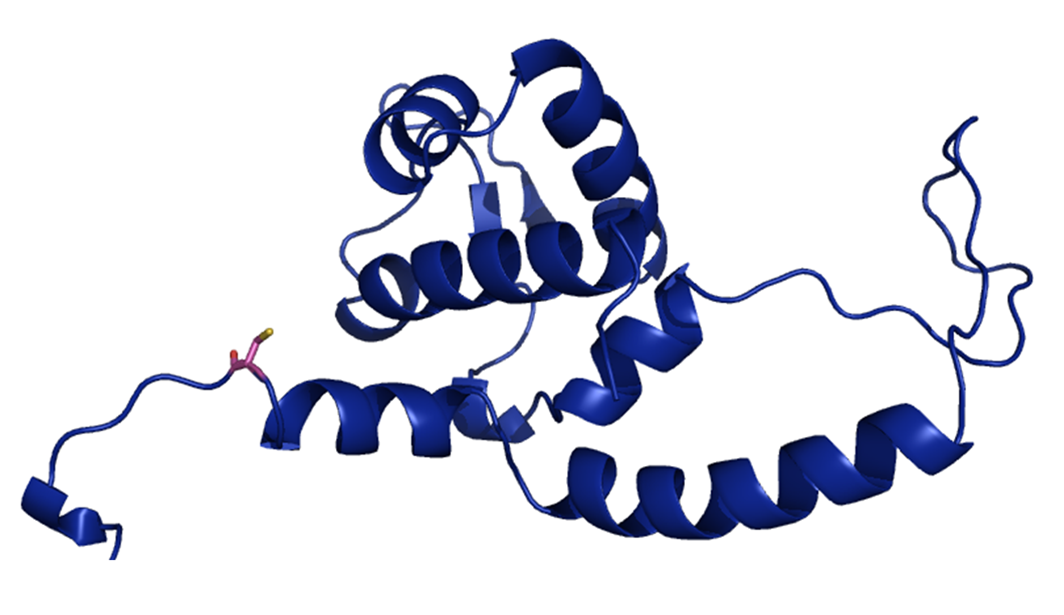

Supplement: Supplementary file 7 — Figure S7. Homology model of the IscR monomer calculated using the MODELLER program. The cartoon representation in blue of the IscR protein shows a helix-turn-helix fold. The surface accessible residue Cys-142 is represented as a stick model in magenta. [file mbo30004-0790-sd7.tif]
