# Supplementary material for: IscR of Rhodobacter sphaeroides functions as repressor of genes for iron-sulfur metabolism and represents a new type of iron-sulfur-binding protein
Source: Microbiologyopen. 2015 Aug 1;4(5):790–802. doi: 10.1002/mbo3.279 (PMC4618611; doi:10.1002/mbo3.279)
Supplement: Supplementary file 8 — Table S1. Rhodobacter sphaeroides and Escherichia coli strains used in this study. Table S2. Plasmids used in this study. Table S3. Oligodeoxynucleotide used in this study. [file mbo30004-0790-sd8.pdf]

Table S1. *R. sphaeroides* and *E. coli* strains used in this study

Strains	Relevant features	References
R. sphaeroides		
2.4.1	*Rhodobacter sphaeroides* wild type	(van Niel, 1944)
2.4.1Δ*iscR*	Sp^r, *iscR* deletion strain	This study
2.4.1Δ*iscR*_pBBR*iscR*	2.4.1Δ*iscR* harbouring pBBR*iscR*	This study
2.4.1-pBE::P_{iscR}::eCFP	Km^r, 2.4.1 containing pBE::P_{iscR}::eCFP, used for *in vivo* reporter system	This study
2.4.1Δ*iscR*-pBE::P_{iscR}::eCFP	2.4.1Δ*iscR* containing pBBR*iscR*, Km^r, used for *in vivo* reporter system	This study
E. coli		
JM109	Host strain for cloning procedures	(Yanisch-Perron et al., 1985)
S17-1	Strain for diparental conjugation, tra^+	(Simon et al., 1986)
M15	Strain for recombinant protein expression	Qiagen
M15-pQE30::*iscR*	M15 containing pQE30::*iscR*, Ap^r, used for IscR overexpression	This study
M15-pQE30::*iscR*_H93A	M15 containing pQE30::*iscR*_H93A, Ap^r, used for IscR overexpression	This study
M15-pQE30::*iscR*_H121A	M15 containing pQE30::*iscR*_H121A, Ap^r, used for IscR overexpression	This study
M15-pQE30::*iscR*_H127A	M15 containing pQE30::*iscR*_H127A, Ap^r, used for IscR overexpression	This study
M15-pQE30::*iscR*_C142A	M15 containing pQE30::*iscR*_C142A, Ap^r, used for IscR overexpression	This study
M15-pQE30::*iscR*_P143A	M15 containing pQE30::*iscR*_P143A, Ap^r, used for IscR overexpression	This study
M15-pQE30::*iscR*_H93A/H121A/H127A	M15 containing pQE30::*iscR*_H93A/H121A/H127A, Ap^r, used for IscR overexpression	This study

Sp^r, spectinomycin-resistant; Ap^r, ampicillin-resistant; Km^r, kanamycin resistant; when required, antibiotics were used in the following concentrations: spectinomycin (10 $\mu g \cdot ml^{-1}$) and kanamycin (25 $\mu g \cdot ml^{-1}$) for *R. sphaeroides*; ampicillin (200 $\mu g \cdot ml^{-1}$) for *E. coli*

Table S2. Plasmids used in this study

Plasmid names	Relevant features	Source
pBBR1-MCS-2	Km^r, broad-host-range cloning vector	(Kovach et al., 1995)
pBBR*iscR*	Km^r, pBBR1-MCS-2 containing *iscR* gene	This study
pET28(a)	Km^r, high expression vector	Novagen
pBE	Km^r, Derivate of pBBR1-MCS-2	This study
pBE4352	Km^r, pBE containing RSP_4352 promoter	This study
pBE4352::eCFP:eCFP	Km^r, pBE4352 containing two eCFP	This study
pBE::P_{iscR}::eCFP	Km^r, pBBR containing *iscR* fragment for *cfp* fusion	This study
pHP45Ω	Sp^r, source of Ω-Sp^r cassette	(Prentki et al., 1991)
pPHU281	Tc^r, *lacZ' mob*(RP4)	(Hubner et al., 1991)
pPHUΔ2.4.1*iscR*	Tc^r, pPHU281 containing *iscR* gene with flanking sites	This study
pPHUΔ2.4.1*iscR*::ΩSp	Tc^r, Sp^r, pPHU281Δ2.4.1*iscR* containing spectinomycin cassette	This study
pJET1.2	Ap^r, 2.97 kb, PCR cloning vector	Fermentas
pQE30	Ap^r, 3.4 kb, 6xHis-tag overexpression vector	Qiagen
pQE30::*iscR*	Ap^r, pQE30 containing *iscR* fragment for overexpression	This study
pQE30::*iscR*_H93A	Ap^r, pQE30::*iscR* containing a mutation of amino acid 93	This study
pQE30::*iscR*_H121A	Ap^r, pQE30::*iscR* containing a mutation of amino acid 121	This study
pQE30::*iscR*_H127A	Ap^r, pQE30::*iscR* containing a mutation of amino acid 127	This study
pQE30::*iscR*_H93A/H121A/ H127A	Ap^r, pQE30::*iscR* containing mutations of amino acids 93, 121 and 127	This study
pQE30::*iscR*_C142A	Ap^r, pQE30::*iscR* containing a mutation of amino acid 142	This study
pQE30::*iscR*_P143A	Ap^r, pQE30::*iscR* containing a mutation of amino acid 143	This study

Sp^r, spectinomycin-resistant; Ap^r, ampicillin-resistant; Tc^r, tetracycline-resistant; Km^r, kanamycin resistant

Table S3. Oligonucleotides used in this study

Name	Sequence	Purpose
RT_RSP_0040_A	TCGAACGACACCAACACC	Forward primer for RSP_0040 (*fliS*) real-time RT-PCR
RT_RSP_0040_B	CACCTTCATGCCGTTGAA	Reverse primer for RSP_0040 (*fliS*) real-time RT-PCR
RT_RSP_0257_A	ACACCTACGGCAACTTCC	Forward primer for RSP_0257 (*pufL*) real-time RT-pCR
RT_RSP_0257_B	ATCGAGTAGCCGACCAGA	Reverse primer for RSP_0257 (*pufL*)) real-time RT-PCR
RT_RSP_0288_A	CGATCAGGCGGTGGTGGT	Forward primer for RSP_0288 (*bchL*) real-time RT-PCR
RT_RSP_0288_B	CGTCGAGGTCCGGCATGT	Reverse primer for RSP_0288 (*bchL*) real-time RT-PCR
RT_RSP_0443_A	GGTGGAAGAGACGCTCAA	Forward primer for RSP_0443 (*iscR*) real-time RT-PCR
RT_RSP_0443_B	ATAGACATGCGCCGAGAC	Reverse primer for RSP_0443 (*iscR*) real-time RT-PCR
RT_RSP_0920_A	GCGAGCTTCGAGGAACTG	Forward primer for RSP_0920 (*exbB*) real-time RT-PCR
RT_RSP_0920_B	GTCTGGCTCTGCGAGATG	Reverse primer for RSP_0920 (*exbB*) real-time RT-PCR
RT_RSP_1545_A	CTTCGAGCGCGAGAT	Forward primer for RSP_1545 real-time RT-PCR
RT_RSP_1545_B	GCCGCAGGAACATCG	Reverse primer for RSP_1545 real-time RT-PCR
RT_RSP_1547_A	GTCTGCCACTGCATGGGCAT	Forward primer for RSP_1547 (*bfd*) real-time RT-PCR
RT_RSP_1547_B	GTTCATTCCTGCGGTTGTGG	Reverse primer for RSP_1547 (*bfd*) real-time RT-PCR
RT_RSP_1669_A	ATCGCGGAAGAGACCCAGAG	Forward primer for RSP_1669 (*rpoZ*) real-time RT-PCR
RT_RSP_1669_B	GAGCAGCGCCATCTGATCCT	Reverse primer for RSP_1669 (*rpoZ*) real-time RT-PCR
RT_RSP_2913_A	CATCAGCCTCGGCAACAC	Forward primer for RSP_2913 (*afuA*) real-time RT-PCR
RT_RSP_2913_B	TGGTTCGTCTCGGCGTAG	Reverse primer for RSP_2913 (*afuA*) real-time RT-PCR
RT_RSP_3416_A	CGAGATGATCCGGCT	Forward primer for RSP_3416 real-time RT-PCR
RT_RSP_3416_B	GTCGGTCGCGGAATA	Reverse primer for RSP_3416 real-time RT-PCR
RT_RSP_3417_A	AGACACGCAAGAGCG	Forward primer for RSP_3417 real-time RT-PCR
RT_RSP_3417_B	GCCACCGATTCCACA	Reverse primer for RSP_3417 real-time RT-PCR
RT_RSP_4275_A	ACACCTCCTTCAGCT	Forward primer for RSP_4275 (*fecI*) real-time RT-PCR
RT_RSP_4275_B	AGACGCAGGAAGATG	Reverse primer for RSP_4275 (*fecI*) real-time RT-PCR
RT_RSP_6006_A	ATGACCGACCCGATGGAG	Forward primer for RSP_6006 (*hemP*) real-time RT-PCR
RT_RSP_6006_B	AGTAGATCTGCCCGTCGAG	Reverse primer for RSP_6006 (*hemP*) real-time RT-PCR
RT_RSP_6020_A	CGGCGAGGTCAAGGTGAT	Forward primer for RSP_6020 (*feoA2*) real-time RT-PCR
RT_RSP_6020_B	TGCAGCGGAGTGACGAAG	Reverse primer for RSP_6020 (*feoA2*) real-time RT-PCR
*suf*1RT_A	CGAACTGCACGGCTTCACCT	Forward primer for RT-PCR
*suf*1RT_B	AGGCGCCATTGTCGAGATAG	Reverse primer for RT-PCR
*suf*2RT_A	CGACCTCGAGGCGCGGATCAAAGG	Forward primer for RT-PCR
*suf*2RT_B	GCGAAGTCCATCTCGATCTCGGTTTC	Reverse primer for RT-PCR
*suf*3RT_A	CTCGGAGAGGAAGAGGCCGGCTC	Forward primer for RT-PCR
*suf*3RT_B	GTGCCGACCGAGACCGAGTCGAAG	Reverse primer for RT-PCR
*suf*4RT_A	AGACGCAGGTGATGCAGATG	Forward primer for RT-PCR
*suf*4RT_B	GAACCGCAGTCTCTTCCAGT	Reverse primer for RT-PCR
*suf*5RT_A	TCTCGGCGCATGTCTATGTC	Forward primer for RT-PCR
*suf*5RT_B	CGTCGCATTCCAGTCGAGAT	Reverse primer for RT-PCR
0443_upA	GAGCCGCCGAATTCCGGGTGC	Forward primer for RSP_0443 cloning
0443_upB	GAGCGCGGGATCCACCAGCG	Reverse primer for RSP_0443 cloning
0443_downA	GCGTGCAGGATCCTTGACCAACCG	Forward primer for RSP_0443 cloning
0443_downB	GAGACAGCCTCAAGCTTCACGTCG	Reverse primer for RSP_0443 cloning
IscR_complA	TAATCTAGAACCATTCCACCTGGGCG	Forward primer for RSP_0443 complementation
IscR_complB	CGCTGGATCCTTCACTCTGGC	Reverse primer for RSP_0443 complementation
IscR-His_fwd	GGCGGGAACCGGGGATCCAAACTCTC	Forward primer for IscR overexpression
IscR-His_rev	CGACAGCCAAGCTTTCAGTCCTCGTC	Forward primer for IscR overexpression
IscR_repA	TCTAGAAAATCACTTCGGGCATCGC	Forward primer RSP_0443 for *in vivo* reporter system
IscR_repB	GGATCCTTTCCGGTTCCCGCCAAATC	Reverse primer RSP_0443 for *in vivo* reporter system
IscR_H93A_A	GCGATGGCTACCGGTGCTGGTGCAAGTG	Forward primer inserting a mutation to H93
IscR_H93A_B	GAGACGGTCAATGCGATGGCTACCGGTG	Reverse primer inserting a mutation to H93
IscR_H121A_A	CTCGGCGGCTGTCTATGTCTTCCTGCAC	Forward primer inserting a mutation to H121
IscR_H121A_B	GTGGGAGGGCCTCTCGGCGGCTGTCTAT	Reverse primer inserting a mutation to H121
IscR_H127A_A	CTTCCTGGCCCAGACCCGTCTGTCGGAC	Forward primer inserting a mutation to H127
IscR_H127A_B	GCATGTCTATGTCTTCCTGGCCCAGACC	Reverse primer inserting a mutation to H127
IscR_C142A_A	GCGTCCAGCCCCGGCGGTGCCGGCGCTG	Forward primer inserting a mutation to C142
IscR_C142A_B	CAAAAACGAGATGCGTCCAGCCCCGGCG	Reverse primer inserting a mutation to C142
IscR_P143A_A	GCGTCCAGCCCCGGCGGTGCCGGCGCTG	Forward primer inserting a mutation to P143
IscR_P143A _B	AAACGAGATGCGTCCATGCGCGGCGGTG	Reverse primer inserting a mutation to P143
*iscR*_up_fwd	CGCGGCGTAATCTTGACAAAAACG	Forward primer for generation of the *iscR* promoter region
*iscR*_up_rev	CGACACGTCGACAAGCGAGACAAG	Reverse primer for generation of the *iscR* promoter region
*hemP*_up_fwd	CGCATAAGTCGACCGAAAGAATCAG	Forward primer for generation of the *hemP* promoter region
*hemP*_up_rev	CCGTCGACAAGGATCCGGGCC	Reverse primer for generation of the *hemP* promoter region

References

HUBNER, P., WILLISON, J. C., VIGNAIS, P. M. & BICKLE, T. A. 1991. Expression of regulatory nif genes in *Rhodobacter capsulatus*. *J Bacteriol,* 173**,** 2993-9.

KOVACH, M. E., ELZER, P. H., HILL, D. S., ROBERTSON, G. T., FARRIS, M. A., ROOP, R. M., 2ND & PETERSON, K. M. 1995. Four new derivatives of the broad-host-range cloning vector pBBR1MCS, carrying different antibiotic-resistance cassettes. *Gene,* 166**,** 175-6.

PRENTKI, P., BINDA, A. & EPSTEIN, A. 1991. Plasmid vectors for selecting IS1-promoted deletions in cloned DNA: sequence analysis of the omega interposon. *Gene,* 103**,** 17-23.

SIMON, R., O'CONNELL, M., LABES, M. & PUHLER, A. 1986. Plasmid vectors for the genetic analysis and manipulation of rhizobia and other gram-negative bacteria. *Methods Enzymol,* 118**,** 640-59.

VAN NIEL, C. B. 1944. The Culture, General Physiology, Morphology, and Classification of the Non-Sulfur Purple and Brwon Bacteria. *Bacteriol Rev,* 8**,** 1-118.

YANISCH-PERRON, C., VIEIRA, J. & MESSING, J. 1985. Improved M13 phage cloning vectors and host strains: nucleotide sequences of the M13mp18 and pUC19 vectors. *Gene,* 33**,** 103-19.
